# Cost-effectiveness of contrast-enhanced breast MRI in suspicious mammographic microcalcifications

**DOI:** 10.1186/s13244-025-01990-y

**Published:** 2025-06-12

**Authors:** Barbara J. Fueger, Fabian Tollens, Clemens G. N. Kaiser, Thomas H. Helbich, Nina Pötsch, Paola Clauser, Pascal A. T. Baltzer

**Affiliations:** 1 Division of General and Pediatric Radiology, Department of Biomedical Imaging and Image-Guided Therapy, Medical University of Vienna, Vienna General Hospital, Vienna, Austria; 2 Department of Radiology and Nuclear Medicine, University Medical Centre Mannheim, Medical Faculty Mannheim, University of Heidelberg, Heidelberg, Germany

**Keywords:** Breast cancer, Screening, Biopsy, Shared decision making, Cost-effectiveness analysis

## Abstract

**Objectives:**

To evaluate the cost-effectiveness of supplemental breast magnetic resonance imaging (MRI) in women with BI-RADS 4 mammographic microcalcifications in order to avoid unnecessary stereotactic biopsies.

**Methods:**

Decision analysis and Markov modeling were used to compare the short-term costs and effects of two diagnostic strategies: supplemental breast MRI in women with mammographic microcalcifications to avoid needle biopsies in MRI negative cases vs stereotactic biopsies of all BI-RADS 4 microcalcifications.

**Results:**

Applying supplemental breast MRI resulted in comparable costs and outcomes. Average cumulative costs of US$ 56,918 and 2.932 quality adjusted life years (QALYs) per woman were achieved for the supplemental breast MRI-strategy, whereas stereotactic biopsies as standard of care resulted in cumulative costs of US$ 56,898 and 2.930 QALYs, resulting in an incremental cost effectiveness ratio (ICER) of US$ 10,047 per QALY gained.

**Conclusion:**

Due to comparable diagnostic safety at similar costs, the non-invasive breast MRI alternative for workup of mammographically detected suspicious calcifications should be offered to patients within the context of shared decision making.

**Critical relevance statement:**

Contrast-enhanced MRI of the breast should be offered as an alternative to stereotactic biopsy within the context of shared decision-making.

**Key Points:**

- Breast MRI and stereotactic biopsy enable accurate risk stratification of suspicious calcifications.
- Breast MRI and stereotactic biopsy yield comparable cost-effectiveness and clinical outcomes.
- Breast MRI should be considered as an option regarding shared clinical decision-making.

**Graphical Abstract:**

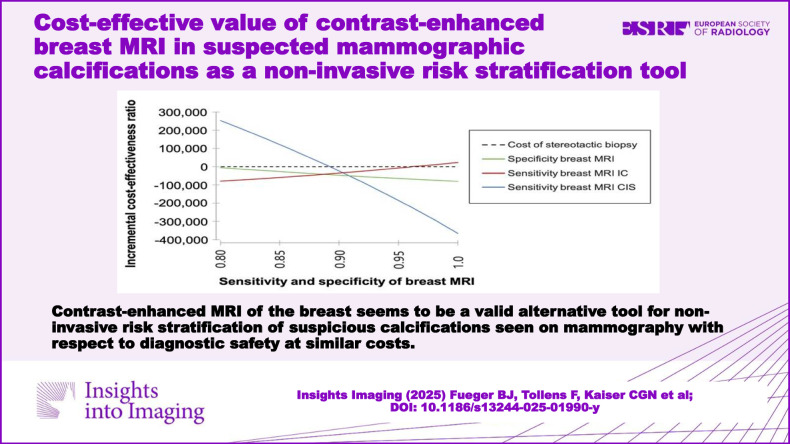

## Introduction

On screening mammography, microcalcifications are seen in up to 31% of the examinations [1]. Malignancy rates vary between 6% and 82%, depending on their imaging characteristics on mammography [2]. In case of suspicious calcifications classified as Breast Imaging Reporting and Data System (BI-RADS) 4 or 5, stereotactic-guided vacuum-assisted breast biopsy (VABB) or large core needle biopsy is recommended to exclude malignancy [3]. Although stereotactic guided VABB is considered safe, minor complications, including pain and scarring at the site of the biopsy, have been reported [4]. Furthermore, the psychological and physical pain due to the procedure should not be neglected [5].

Growing evidence indicates that contrast-enhanced magnetic resonance imaging (CE-MRI) of the breast might be feasible and accurate to assess these calcifications [6–8]. A recent meta-analysis reported that a negative CE-MRI of the breast could potentially avoid 80.6% of unnecessary biopsies in microcalcifications classified as BI-RADS 4 at a cost of 3.7% missed cancers [9], with a percentage of missed invasive cancers (ICs) in 1.6% [9].

By switching management to imaging surveillance rather than invasive diagnostic procedures, a significant number of biopsies could be avoided. Stereotactic guided VABB, as well as supplemental breast MRI, are afflicted with significant costs to the healthcare system. False-positive findings need further work-up and increase the costs [10, 11].

In addition to the diagnostic performance, a comprehensive assessment of costs and downstream effects is required by healthcare decision makers to estimate the value of a new technique. Cost-effectiveness analyses have become an accepted methodology to prove the value of innovative diagnostic procedures from an economic point of view [12].

The aim of this economic evaluation was to assess the cost-effectiveness of CE-MRI in comparison to stereotactic guided VABB in suspicious—BI-RADS 4—calcifications seen on mammography. To determine whether VABB or breast MRI followed by mammography is more cost-effective is an important aspect of health economics in breast imaging and has not been evaluated so far. To the best of our knowledge, no studies have been published regarding this important topic.

## Material and methods

This study used study-level results and was exempt from institutional review board approval at our university. Due to the retrospective nature of the data used, a distinct health economic analysis plan was not prepared. All relevant information is contained in this section.

### Cost-effectiveness modeling

#### Decision model and diagnostic strategies

Two diagnostic strategies were included in the decision tree: as standard of care, women with suspicious microcalcifications (BI-RADS 4) received stereotactic VABB to rule out or confirm malignancy. The alternative strategy included a CE-MRI examination to further characterize the breast lesions. Only in the case of a persistently suspicious lesion (BI-RADS 4) did women receive stereotactic VABB of the calcifications.

When downgrading the lesions to BI-RADS 3 by breast MRI, an invasive biopsy would be avoided, and mammographic follow-up at 12 months and 24 months would be conducted.

**Fig. 1:**
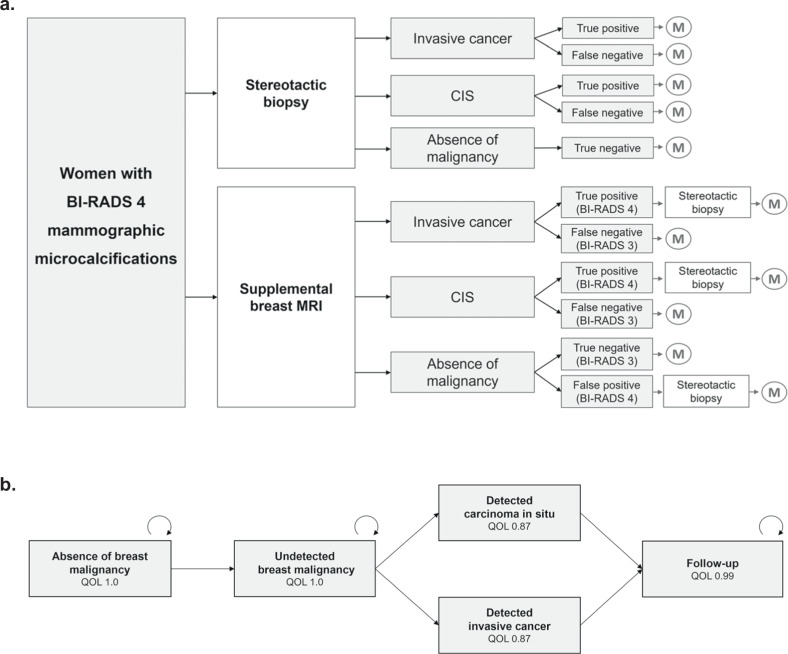
Cost-effectiveness analysis and modeling. **a** Decision tree comprising two alternative strategies. Every positive finding in breast MRI is followed by a stereotactic biopsy, whereas every negative finding in breast MRI does not need to get a biopsy, but is followed up by X-ray mammography after 12 months or 24 months. **b** Markov model with a cycle length of one month and a time horizon of 36 months. Mortality is simulated for each stage (not depicted). Quality of life (QoL) is defined for each Markov state. CIS, cancer in situ; M, mammography; QoL, quality of life

To compare the two diagnostic strategies, a decision tree was designed that incorporated the possible outcomes true positive, false positive, true negative, and false negative, except for false positive biopsy findings that are, by definition, an impossible outcome (Fig. 1a).

#### Markov outcome model

A Markov model was developed to estimate the short-term costs and outcomes of the two aforementioned diagnostic strategies. A model runtime of 36 months with a cycle length of one month was selected. Markov states reflected the ground truth and included the absence of breast malignancy, undetected breast malignancy, accurately detected breast malignancy, including therapy, and follow-up (Fig. 1b).

Quality of life (QoL) was estimated for each Markov state based on literature and on a few assumptions, such as 100% specificity of VABB, a QoL of 1.0 for absence of cancer, 0.99 for patients in follow-up, −0.05 for patients undergoing an unnecessary biopsy and 0 for dead patients [13]. Biopsies that ruled out breast malignancy were considered potentially avoidable and resulted in impaired QoL. Cancer treatment, as well as stereotactic biopsies, resulted in reduced QoL (Table 1).

**Table 1:** Input parameters for the economic modeling

Variable	Estimation	Source
Cancer prevalence (IC and CIS)	43.6%	(9)
Proportion of CIS	41.9%	(9)
Average age	53	(9)
Incidence of breast cancer	Age-specific incidence rates	SEER age-adjusted incidence rates 2017 (32)
Assumed WTP	$ 100,000	(20)
Discount rate	3.00%	(20)
Diagnostic test performances		
Sensitivity of stereotactic biopsy	95.7%	(33, 34)
Specificity of stereotactic biopsy	100.0%	Assumption
Sensitivity of MRM		
IC	−97.9%	
Carcinoma in situ	−88.9%	(9)
Specificity of MRM	77.0%	(9)
Costs		
XM	$127.82	Medicare (CPT code 77067)
MRM	$366.00	Medicare (CPT code C8906)
Stereotactic biopsy	$1700.00	Medicare (CPT code 19081)
Cost of treatment		
IC	−$140,511	
Carcinoma in situ (inflated to 2024 $)	−$103,545	(35)
Utilities		
QoL of patients without a detected tumor	1.00	Assumption
QoL of patients with detected tumor (during treatment)	0.87	(14)
QoL of patients in follow-up	0.99	Assumption
Reduction in QoL due to avoidable biopsy	−0.05	Assumption
Death	0.00	Assumption
Transition probabilities		
Average annual death rate	Age adjusted	US Life Tables 2017, women of all ethnicities (19)

Model assumptions: follow-up by mammography at 12-month intervals in case of negative MRI, post-therapeutic follow-up by mammography at 12-month intervals, model time horizon: 3 years, cycle length: 1 month

*CIS* cancer in situ, *CPT* current procedural terminology, *IC* invasive cancer, *MRM* MR mammography, *QoL* quality of life mammography, *SEER* surveillance, epidemiology, and end results program, *WTP* willingness to pay, *XM* mammography

Costs were estimated in 2024 US$ based on Medicare Current Procedural Terminology (CPT) codes and recent literature [14] and inflated to 2024 US$ where necessary. Costs and quality-adjusted life years were discounted and summed up over the total model runtime.

### Model input parameters

For this cost-effectiveness study investigating stereotactic biopsy and breast MRI to characterize BI-RADS 4 lesions in screening mammography, relevant input parameters such as diagnostic sensitivity and specificity of the procedures were extracted from recent literature from a systematic review and meta-analysis (Table 1) [15, 16].

#### Diagnostic efficacy parameters

Recently, a comprehensive meta-analysis investigated whether contrast-enhanced breast MRI can avoid biopsies in suspicious microcalcifications seen on mammography [9]. This study reported a pooled rate of avoidable biopsies of 80.6% (95% CI: 64.6–90.5%) while the overall and IC false negative rates were 3.7% (95% CI: 1.2–6.2%) and 1.6% (95% CI: 0–3.6%), respectively. The proportion of carcinoma in situ (CIS) was 41.9%, and the pre-test probability of malignant lesions (IC and CIS) was 43.6%.

#### Utilities

Throughout the model, QoL and the respective time intervals were used to calculate quality-adjusted life years (QALYs). To account for a reduced QoL in patients with a detected tumor (during treatment) 0.87 QALYs per year were assumed [14]. The reduction of QoL of patients in follow-up was assumed with 0.99 QALYs per year, which is based on prior cost-effectiveness analyses [17, 18]. The reduction of QoL due to avoidable biopsies was accounted for with −0.05 QALYs.

#### Cost estimates

**Fig. 2:**
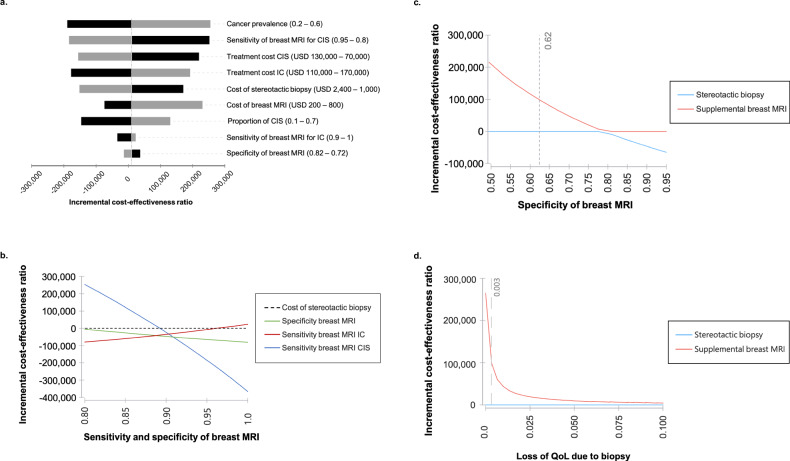
Deterministic sensitivity analysis. **a** The impact of variations of input parameters on the resulting incremental cost ratio (USD/QALY gained) of supplemental breast MRI is depicted in a tornado diagram. Input variables are varied between lower limits (black bar) and upper limits (gray bar) as indicated in the brackets. **b** Variations of sensitivity of breast MRI for IC or CIS or specificity of breast MRI result in different cumulative costs, while holding all other variables constant (see Table 1 for base case assumptions). For instance, assuming a reduced specificity of breast MRI results in higher incremental cost-effectiveness ratios. **c** Varying the specificity of breast MRI, i.e., the rate of avoidable biopsies, affects the cost-effectiveness. **d** Varying the assumed loss in QoL due to stereotactic biopsies affects the cost-effectiveness. The smaller the assumed loss of QoL, the lower the resulting cost-effectiveness of breast MRI. CIS, cancer in situ; IC, invasive cancer; QoL, quality of life; USD, US dollar

Costs of a stereotactic biopsy were calculated based on the current U.S. Medicare (medicare.gov) in the setting of hospital outpatient departments with US$ 1700 (Medicare code 19081) and the costs for a breast MRI at US$ 366 (Medicare code C8906). The range of costs for breast MRI was considered US$ 200–800 and US$ 800–2000 for a stereotactic biopsy. Overall costs of treatment were assumed with US$ 140,511 (110,000–170,000 US$) for IC and US$ 103,545 (70,000–130,000 US$) for CIS, based on [14] and inflated to 2024 US$. The resulting incremental costs were visualized in a tornado plot (Fig. 2a). Particular focus has been laid on the impact of the specificity of breast MRI, which might be smaller due to incidental suspicious findings (Fig. 2b).

#### Transition probabilities

Average age-adjusted mortality rates of women of all ethnicities were included from the U.S. Life Tables [19].

### Cost-effectiveness analysis

Decision analysis and Markov modeling were conducted using specialized software for economic evaluations (TreeAge Pro 2020, TreeAge Software, Williamstown, MA). The perspective of the United States healthcare system was selected for this cost-effectiveness analysis. An annual discount rate of 3% was incorporated for costs and outcomes, which is in line with current recommendations on the methodology of cost-effectiveness analyses [20]. Cumulative discounted costs and outcomes were modeled for each diagnostic strategy.

### Sensitivity analysis

#### Deterministic sensitivity analysis

In order to cope with the uncertainty of the input parameters and to evaluate their impact on the model outcomes, a deterministic sensitivity analysis was applied. In order to examine the impact of varying input parameters on the resulting incremental costs, the diagnostic performance of breast MRI and the costs of the diagnostic procedures were taken from the aforementioned systematic review [9]. Since empirical data on the impact of stereotactic biopsies on the QoL are unavailable, the assumption of an impaired QoL of 0.05 has been examined by an extensive sensitivity analysis.

## Results

### Cost-effectiveness analysis

Conducting supplemental breast MRI in mammographic BI-RADS 4 microcalcifications resulted in higher short-term costs and favorable outcomes for a 12-month follow-up, and smaller short-term costs and favorable outcomes for a 24-month follow-up.

The standard of care using stereotactic biopsies resulted in average cumulative costs of US$ 56,898 for each woman and cumulative effects of 2.930 QALYs with a mammography after 12 months and with a mammography after 24 months in average cumulative costs of US$ 56,359 and cumulative of effects of 2.930 QALYs (Table 2). The alternative strategy applying supplemental breast MRI resulted in average cumulative costs of US$ 56,918 and cumulative effects of 2.932 QALYs per woman with a mammography after 12 months and US$ 56,266 and 2.932 QALYs, respectively, with a mammography after 24 months. For the 12-month follow-up, this results in an incremental cost-effectiveness ratio (ICER) of US$ 10,047 per QALY gained.

**Table 2:** Results of the cost-effectiveness analysis, assuming

(a)				
Strategy	Cumulative discounted costs (USD)	Incremental cost (USD)	Cumulative discounted effects (QALYs)	Incremental effects (QALYs)
Supplemental breast MRI	56,918	–	2.932	–
IC	142,834	–	2.910	–
CIS	109,544	–	2.911	–
Absence of malignancy	1283	–	2.948	–
Stereotactic biopsy	56,898	20	2.930	−0.002
IC	142,435	−399	2.910	0.000
CIS	107,078	−2466	2.910	−0.001
Absence of malignancy	2226	943	2.945	−0.003
(**b**)				
Supplemental breast MRI	56,266	–	2.932	–
IC	142,477	–	2.910	–
CIS	107,655	–	2.912	–
Absence of malignancy	900	–	2.948	–
Stereotactic biopsy	56,359	93	2.930	−0.002
IC	141,695	−782	2.910	0.000
CIS	106,338	−1317	2.910	−0.002
Absence of malignancy	1843	943	2.945	−0.003

(a): a 12-month and (b): 24-month follow-up by mammography after a negative MRI

*CIS* cancer in situ, *QALY* quality-adjusted life year, *USD* US dollar

### Sensitivity analysis

Variations in the costs of the diagnostic procedures and the diagnostic performance of breast MRI resulted in varying costs. Costs of biopsy and specificity of breast MRI were identified as key determinants, and results are summarized in a Tornado plot (Fig. 2a). When the cost per biopsy was greater than US$ 1311, the ICER was below a threshold of US$ 100,000 per QALY.

Varying the specificity of breast MRI between 62% and 100% consistently resulted in ICER values below a threshold of US$ 100,000 per QALY (Fig. 2b).

**Fig. 3:**
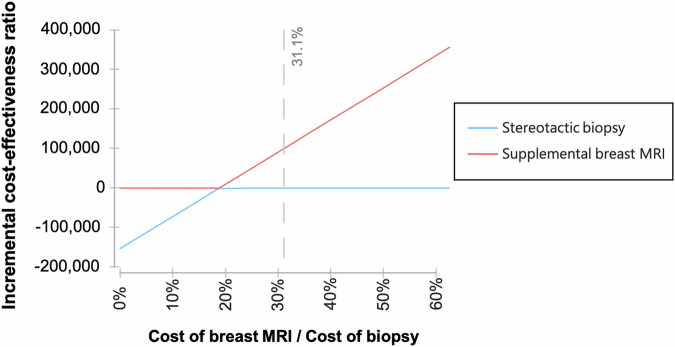
Impact of the costs of breast MRI and stereotactic biopsy on the resulting ICER (in USD/QALY gained). As long as the costs per examination of breast MRI are smaller than 31.1% of the cost of a stereotactic biopsy, applying supplemental breast MRI is cost-effective

Since the cost per examination of breast MRI and the cost of a stereotactic biopsy were identified as major determinants of cost-effectiveness, the relation of these costs and their impact on overall cumulative costs was further examined (Fig. 3). As long as the costs per examination of breast MRI did not exceed 31.1% of the cost of a stereotactic biopsy, applying supplemental breast MRI was cost-saving compared to the standard of care. A negative breast MRI result could downgrade a pooled rate of 76% of mammographic BI-RADS 4 lesions.

## Discussion

We present a cost-effectiveness analysis comparing two diagnostic strategies for managing suspicious microcalcifications classified as BI-RADS 4 on mammography. The current standard to manage BI-RADS 4 calcifications is stereotactic-guided VABB. We compared this with CE-MRI followed by VABB for MRI-positive findings. For MRI negative findings, a follow-up by conventional mammography at 12 and 24 months was used. Cost-effectiveness revealed comparative costs of the primarily invasive and the primarily non-invasive approach.

Many experts and several major medical societies recommend that women at average risk of breast cancer begin screening mammography at some point between the ages of 40 and 50 and continue regular screenings thereafter. Out of all the lesions detected in women receiving screening mammography, approximately 31% of these detected lesions are calcifications [1]. These calcifications need to be further examined because up to 30% turn out to be cancer [21, 22]. The rate of ICs compared to all malignant lesions was about 50% [9]. This implies that a large number of women worldwide are affected, and cost-effectiveness is therefore an important socio-economic aspect in many countries.

Stereotactic-guided VABB is considered an accurate and safe procedure in comparison to open surgery. Adverse events are reported but considered rare, and include hematoma, bleeding, and infection [23]. VABB is considered rather costly; Medicare lists the costs of stereotactic biopsy at US$ 1,700. In comparison, CE-MRI of the breast is cheaper and is listed at US$ 366. Because of the sheer number of women requiring further work-up of mammographically detected microcalcifications, alternative diagnostic approaches are welcome. CE-MRI of the breast using intravenous Gadolinium-based contrast media, offers a less invasive alternative [9, 24] and is more readily available than centers performing stereotactic guided VABB.

We demonstrated that conducting CE-MRI of the breast as a supplemental diagnostic test for BI-RADS 4 microcalcifications seen on mammography results in comparable costs and outcomes, also when follow-up mammography at 12 or 24 months is integrated.

CE-MRI of the breast can play a decisive role in distinguishing benign from malignant BI-RADS 4 calcifications [9]. IC can largely be excluded by contrast-enhanced breast MRI with a negative predictive value (NPV) of about 99%. The sensitivity of contrast-enhanced breast MRI is not as high for ductal carcinoma in situ (DCIS), with a false-negative rate of 3.7%. However, the progression probability for DCIS is 18–50% within the next 5–10 years, providing ample opportunity to clinically react within a follow-up focused approach [25]. While the current analysis focused on all women, Fueger et al suggested that BI-RADS 3 benchmarks of a 2% malignancy rate apply after a negative MRI scan in case of pretest probabilities of up to 22%. This means that the suggested alternative approach of breast MRI instead of immediate VABB is best suited for BI-RADS 4a and some 4b calcifications, and rather not in the case of BI-RADS 4c. The impact and outcome of potential FN, however, are unknown, and thus, conclusions on whether using breast MRI in BI-RADS 4c cases is potentially unsafe cannot be provided. It is further beyond the scope of this article to recommend whether patients with negative MRI can go back to screening after showing no imaging progress on follow-up due to the lack of specific empirical evidence. We further believe that it is societies and other health policy makers that should provide practice recommendations based on evidence, such as that provided within this article.

Previous studies have explored the cost-effectiveness of CE-MRI of the breast in different contexts, such as screening patients at intermediate risk of breast cancer and women with dense breasts [17, 26, 27]. However, to our knowledge, no cost-effectiveness analysis has specifically evaluated CE-MRI of the breast as a substitute for immediate VABB in BI-RADS 4 microcalcifications. This study addresses this gap, demonstrating that CE-MRI of the breast can avoid a significant number of > 70% of unnecessary biopsies, while maintaining acceptable costs related to false-negative results.

The current analysis is based on published data (sensitivity and specificity) that also covers false-positive findings. Indeed, the available data are insufficient to assess whether false positive findings were incremental and discrepant with false positive findings on mammography. Thus, the case may arise where a more expensive MRI-guided biopsy is required instead of stereotactic-guided VABB. Considering the costs of both and our own clinical practice, we deem a large number of MRI-only lesions unlikely. In addition, MRI-identified lesions for the most part undergo US-guided procedures, which are more available and less expensive than stereotactic procedures. MRI follow-up for patients with false-positive results is a highly interesting point that should be investigated in detail in a future analysis focusing on MRI strategies.

Our analysis is based on a systematic review and meta-analysis, indicating that 80.6% of VABBs in BI-RADS 4 cases could be avoided with breast MRI, at the cost of a 3.7% missed breast cancer rate, 1.6% of which are invasive. This suggests that CE-MRI of the breast provides a diagnostically valid alternative to immediate biopsy, reducing invasive procedures without significantly compromising diagnostic accuracy.

Sensitivity analysis indicates that variations in the sensitivity and specificity of CE-MRI of the breast influence cumulative costs while holding other variables constant. When considering the potential reduction in QoL due to avoidable biopsies, the cumulative quality-adjusted life years (QALYs) differ more significantly between the two strategies. Without accounting for QoL reductions, both strategies yield identical cumulative outcomes of 2.932 QALYs. This highlights the need for empirical data on the QoL impact of different diagnostic pathways.

Our study is model-based and must be interpreted within its limitations. The Markov model, while comprehensive and may include several diagnostic situations, cannot fully replicate clinical reality. However, our model is capable of simulating two of the main diagnostic procedures and takes follow-up with a mammography at 12 months and 24 months into account. The current analysis uses U.S. healthcare system data. Economic evaluations of the U.S. healthcare system (Medicare) have evolved as an international standard due to the excellent availability and transparency of cost data, which is why most studies apply a U.S. healthcare perspective. This facilitates comparisons between studies and with respect to benchmarks. Translation to European countries is challenging due to the number of countries and the heterogeneity of the healthcare systems, with various sub-systems that each have differing cost structures.

Due to the invasive character of stereotactic biopsies, a temporary reduction in QoL by 0.05 was assumed for this analysis. When not assuming a reduced QoL due to unnecessary biopsies, the resulting short-term outcomes of the two alternative diagnostic strategies are equal. This reflects the fact that there is no evidence of increased QoL or survival in the case of an MRI assessment in the study collective up to this point.

Another limitation is false positive findings and additional incidental, potentially malignant findings on CE-MRI of the breast that require additional workup and for which currently no reliable data exist in the investigated setting [28, 29]. The anxiety associated with longer diagnostic pathways, are area that has been demonstrated [30, 31]. It thus should be noted that careful communication and shared decision-making are necessary to provide patients with oncologically safe decisions that also align with the patient’s needs. At comparable costs, immediate biopsy would very much be the preferred method to resolve suspicious mammographic calcifications in case patient compliance regarding follow-up visits is expectedly low or patient anxiety challenges the concept of follow-up visits.

In conclusion, this is the first study to evaluate the cost-effectiveness of CE-MRI of the breast compared to stereotactic guided VABB for suspicious—BI-RADS 4—microcalcifications seen on mammography. Given its diagnostic safety and almost equal costs, CE-MRI of the breast should be considered a viable alternative within the context of shared decision-making with patients.

## Data Availability

We used the following published information as a basis for our analysis: Fueger et al [9] 10.1016/j.breast.2021.02.002.

## Abbreviations

BI-RADS: Breast imaging reporting and data system
CE-MRI: Contrast-enhanced magnetic resonance imaging
CIS: Carcinoma in situ
CPT: Current procedural terminology
DCIS: Ductal carcinoma in situ
IC: Invasive cancer
ICER: Incremental cost-effectiveness ratio
MRI: Magnetic resonance imaging
QALY(s): Quality-adjusted life year(s)
QoL: Quality of life
USD: United States Dollar
VABB: Vacuum-assisted breast biopsy

## Declarations

### Study design

This study employed a decision-analytic model and Markov simulation to evaluate the cost-effectiveness of supplemental contrast-enhanced breast MRI compared to stereotactic VABB in women with BI-RADS 4 mammographic microcalcifications.
